# Expansion of hay production and marketing in Brazil

**DOI:** 10.1016/j.heliyon.2021.e06787

**Published:** 2021-04-16

**Authors:** M.A. Neres, C.D. Nath, S.M. Hoppen

**Affiliations:** Department of Animal Science, State University of West Paraná, Marechal Cândido Rondon, PR, 85960-000, Brazil

**Keywords:** Baler, Conditioner mower, Forage conservation, Haymaking, Supplementary forage, Prismatic bales

## Abstract

In the last decade, advances in Brazilian hay production showed that the country has the potential to produce bulky dehydrated fodder. For many years, the Brazilian hay production scientific knowledge had been based on temperate climate species, even though the best hay material are tropical grasses, as Tifton 85 Bermudagrass. Researches that focused on the comprehension of yield systems, biochemical processes, physiology, composition, and nutritional quality of tropical species under dehydration and conservation have become important to hay yield in Brazil. Therefore, this literature review aimed to discuss the hay research contribution in tropical conditions and its reflex on the production and commercialization of hay in Brazil. This review was based on database research with key-words defined in a period between 1960 and 2021, which resulted in 33 articles. Each article had the strengths and weaknesses, opportunities, and threats classified according to the SWOT matrix. Articles related to the haymaking system with tropical forage and the effects on nutritional value, sanitary quality, and factors that influence the dehydration period in the field and storage were listed in this paper. Based on the literature, the conclusion is that Brazil has elevated hay yield potential with high nutritional and sanitary quality of tropical species due to the weather conditions that allow fast dehydration and, also, the availability of residual wastewater as fertilization and machinery appropriated. Brazilian haymaking and commercialization are in an expansion process with economic return as national and international trade. Further challenges: to obtain a constant annual hay supply and the transport viability to markets distant from the production center.

## 1 Introduction

The feeding system used in animal production in Brazil has, for many years, been based on the use of tropical grass pastures. These tropical fodders have well-defined production seasonality such that producers have to wait for the next growth cycle of C4 plants, which depends on photoperiod, rainfall, and temperature for regrowth and increase in production. During the inter-harvest period, producers adopted the practice of selling animals on property or have waited for favorable weather conditions for the fodder to grow. Therefore, the production of beef cattle was characterized by the expectation of compensatory gain, which is dependent on maintenance and gain or loss of weight during the inter-harvest season.

In recent decades, there has been an increase in roughage supplementation in herds that owe to great awareness among producers of the losses that occur during the inter-harvest period and the adoption of more intensive production systems, where pastures alone do not govern weight gain and milk production.

The silage technique was pioneered in Brazil and was adopted in 1920 using whole corn plant as the main silage fodder (Bernanrdes and Amaral, 2010). This technique was based on the European model, with circular concrete aerial silos as storage structures. From 1960 onwards, the ensiling of elephant grass and sorghum has also been practiced. However, the lack of appropriate harvesting equipment and high moisture content of elephant grass plants at the time of cutting limited the practice; therefore, research was necessary to solve these problems (Lavezzo, 1993).

Other fodders, such as tropical climate grass (Tanzania, Mombasa, Braquiarão, Tifton 85 Bermudagrass, and Coastcross), temperate climate grass (Oat, Ryegrass, and Triticale), sugar cane, and cereal grains have been gaining prominence in silage making since 1990. Their storage structures have also changed from concrete tower silos to bunker silos, which are less expensive and easier to fill, compact, and unload.

Intensification of cattle, goat, sheep, and bubaline production led to conserved fodder production planning to ensure uninterrupted roughage supplementation of these animals. Thus, space was created on properties for silage storage structures and roughage production.

**Figure 1:**
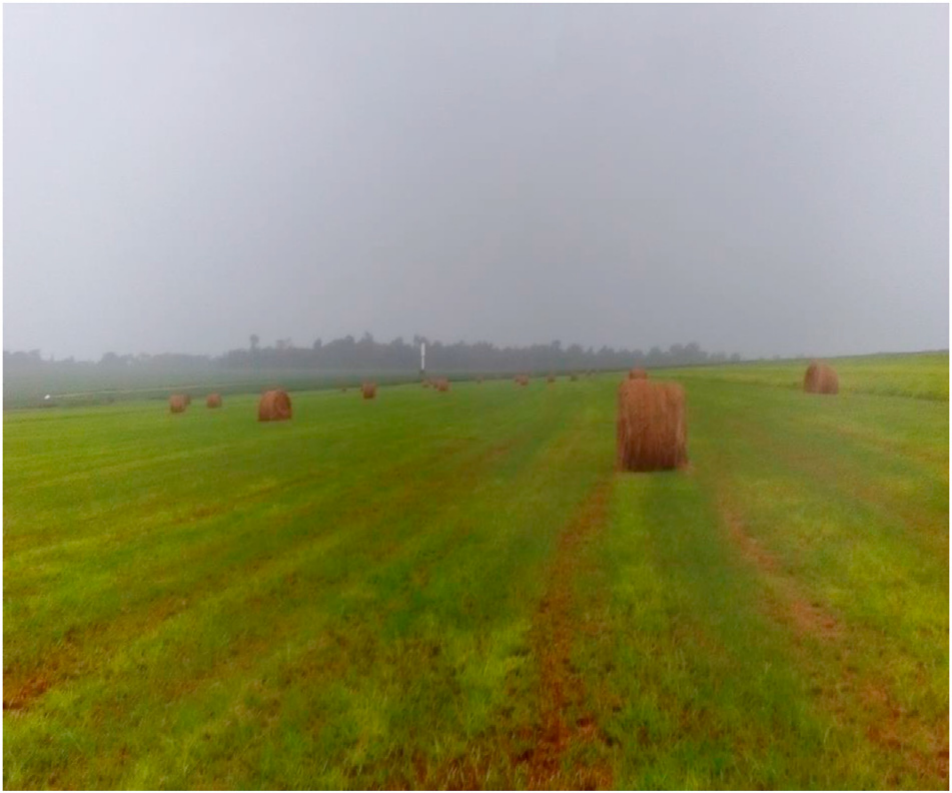
Baled, moist hay caused by precipitation before storage (personal file).

Hay, for roughage supplementation, was adopted late by producers in Brazil. There are reports of its use in 1923 (Geiger and Jablonsky, 1923). However, several factors influenced the rarely use of this technique for hay production, which include: limited regions with a climate suitable for the production of hay, given that the technique requires favorable weather conditions; inadequate investment by producers on equipment and machinery for cutting, turning, windrowing, and baling, and sheds for storage; and lack of instruments for forecasting weather conditions suitable for field drying forage, with a safety margin. Some regions of the country have unfavorable weather for hay production, mainly owing to their high relative air humidity (Figure 1), which prevents hay to reaches ideal levels of dry matter (DM) for adequate conservation, thus resulting in low sanitary quality of roughage and favoring the development of mycotoxins (Rotz and Muck, 1994).

For many years, it was believed that the cost of silage DM was much lower than the cost of hay DM (R$0.28/kg/DM corn silage and R$0.50/kg/DM hay (Tifton 85 Bermudagrass type A - high quality)); this difference in cost is seen throughout the country. However, in regions where fertilization is performed using residual water from swine breeding, the difference in cost is small due to the low cost of hay DM (R$0.30/kg/MS). Another important factor that has contributed to the expansion of haymaking is the distinction that producers currently make between two sources of roughage: corn silage, which is a source of fiber and energy with a moisture content of approximately 65 g/kg, and hay, which is a source of physically effective fiber, with a crude protein (CP) content higher than that of corn silage, depending on species, management, and fertilization.

Since 1985, with the easy access to imported machinery and the interest of Brazilian industries in developing haymaking equipment, there has been an increase in the technification of the hay production system and, consequently, an increase in production. The equipment was imported from the United States, France, England, and Germany, and there were also a few options in the domestic market. However, Brazilian industries started specializing in the production of these implement such that they currently have manufacturing industries, which are more concentrated in the states of São Paulo, Paraná (Castro), Santa Catarina, and the Rio Grande do Sul.

Hay is conserved roughage with low water content (approximately 15 g/kg); thus, its production was intended not only for use on the property but also for sales. This literature review aimed to approach the related aspects to hay technology advances in Brazil and its effects on plant nutritional value, sanitary quality, and physiological responses, as well as the ideal hay storage systems in tropical conditions.

## 2 Methodology

The paper analyses were performed by bibliometry. The articles were obtained in databases, with a selection of publications related to hay yield systems forages dehydration physiology, tropical hay nutritional values, gas exchanges in plants, and hay storage systems. The academic community and network related to the subject were mapped. The period analyzed was from 1960 to 2021, which listed 33 articles. The SWOT was used; it was created by Albert Humphrey over the 60/70 decades, and adapted by Kenneth Andrews and Roland Christensen, to filter the strength and weaknesses, opportunities, and threats of each article (Benzaghta et al., 2021).

The main databases used for this research were: Scopus, Web of Science, scielo.org.br and periódicos.capes.gov.br.

## 3 History of haymaking in the world

Agricultural activity arose when humans made a transition from nomadic to a sedentary lifestyle. Agricultural cultivation (called ager) appeared with the best lands used for growing lupin, flax, barley, einkorn wheat, emmer wheat, chickpea, pea, and vetch, for an average of two consecutive years (Mazoyer and Roudart, 2010). After that period, the cultivation areas were set aside (called alqueive) and grazed at night by cattle to restore soil fertility through animal manure. After two years, the ager area was rotated with the alqueive area. Peripheral zones of a so-called silver system were occupied by native forest, from which wood for firewood and construction of houses destined (Neres et al., 2017).

With an increase in population and, consequently, an increase in the demand for grain production combined with the sharp reduction in soil fertility and winter fodder deficit, farmers started to store part of their surplus fodder produced in the silver areas.

However, due to the hand tools utilization, the haymaking process was slow and time-consuming (BACC II Author Team, 2015). Hay was stored on-site in the form of haycocks. During the cold periods, cattle were taken to peripheral areas for supplementation owing to a lack of fodder for grazing.

During the land rotation system, there was a need to fertilize the alqueive areas with manure to restore the soil fertility. Therefore, animals were separated to spend the night in the alqueive areas. Over the animal transportation from the silver to alqueive, part of the manure was lost, indicating a limitation of the system (BACC II Author Team, 2015). As a result, sheds were used to shelter the animals near the alqueive areas, and the hay storages from silver areas were arranged on wooden covered structures (Spulerová et al., 2019). Herds then spent the entire cold season in stables, which allowed the manure collection at night (Neres et al., 2017).

Over the middle ages, the haymaking process improved with the animal traction introduction or more productive hand tools, such as cutlass. During the contemporary age, there was a great agriculture advance with the steam engine development that culminated in 1892 in gasoline tractors invention in the United States (Vian et al., 2013). From that period onwards, advances occurred in the manufacture of hay implements, which resulted in a faster and more effective production process (Neres et al., 2017).

## 4 Hay production in Brazil: commercialization

Hay is a roughage material that has undergone dehydration in the sun or by artificial means after cutting and is stored at a moisture level below 20% to avoid losses caused by oxidative reactions and microbiological deterioration (Savoie et al., 2011). However, in practice, producers seldom store hay at a moisture level above 15%, to ensure conservation without compromising the quality of the roughage because of the possibility of fermentation processes and the consideration that hay is hygroscopic.

**Figure 2:**
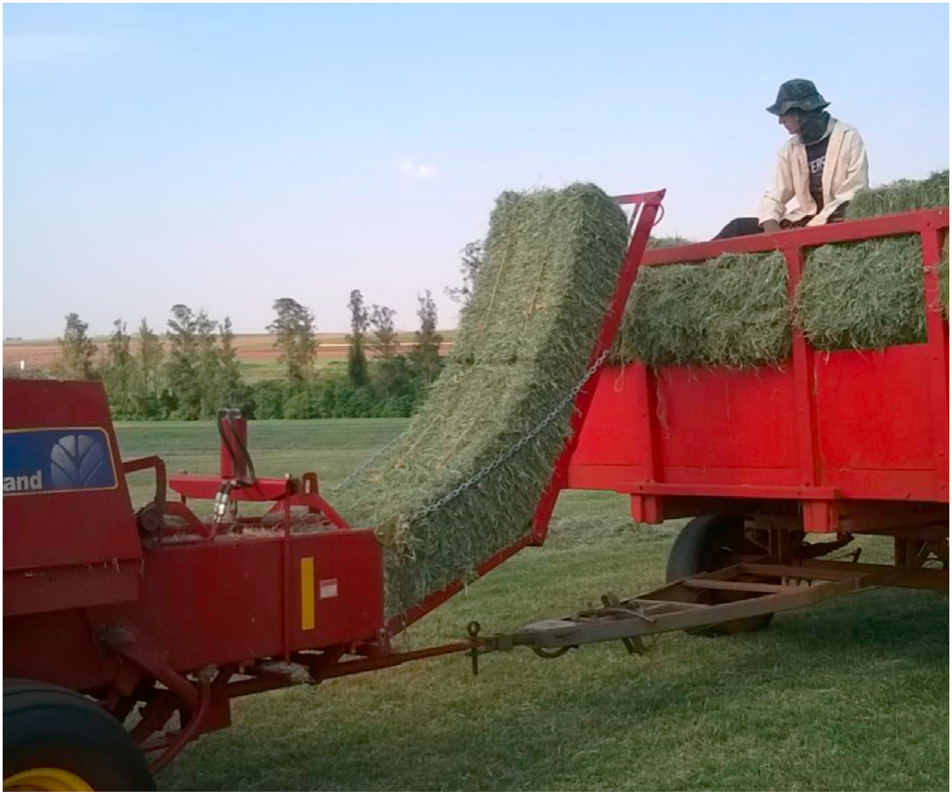
Production of prismatic bales in Marechal Cândido Rondon, State of Paraná (personal file).

In Brazil, hay is mostly commercialized as prismatic bales ranging from 12 kg to 20 kg (Figure 2). It is the preferred form among small- and medium-sized producers because low-weight prismatic bales are practical units for movement on the property. In other words, they are easy to unload, stack in the storage shed, and remove for daily supply to animals. Hay is also commercialized as cylindrical bales, with a weight ranging from 100 kg to 200 kg, but requires specific equipment for loading and unloading.

Few producers have adopted the technique of drying hay in sheds because of the high cost. A part of the properties that have drying sheds produces legume hay, mostly alfalfa. In the last 20 years, Castro (Paraná State) is the main pre-dried hay production region, because its climate is unfavorable for hay production owing to high air relative humidity.

This technique is now used by some hay producers, as it requires half the time of sun exposure for baling and wrapping fodder. Even in regions with a favorable climate, producers have round balers and bale wrappers as alternatives to the small prismatic baler. Thus, when weather conditions do not allow 48 to 36 h of exposure to favorable conditions in the spring/summer or 72 h in the winter (Ames et al., 2014), producers make pre-dried hay in the short weather windows favorable to drying.

In spring/summer, fine-stemmed forage such as Tifton 85 Bermudagrass, when cut in the morning after dew, is baled and wrapped in the afternoon, after approximately 5–6 h of exposure to the sun (Nath et al., 2018).

Producers in the Northern Region of the country, such as Amazonas, Pará, Rondônia, and the Roraima States, do not use the haymaking technique because their weather, including high rainfall, is unfavorable for growth and production of pasture throughout the year. In other states, there are hay produce centers for herds and/or sell to others who prefer to buy from third parties instead of producing. Some locations that do not have favorable weather conditions for hay production purchase from third parties. This territorial advance is due to the mastery of production techniques and access to more accurate and timely weather conditions information by producers. The use of more efficient equipment in terms of speed of operations and the acquisition of mower-conditioners that, through the damage they cause to stems, accelerate the dehydration of plant materials, reducing the number of hours required to achieve complete dehydration.

Hay for storage needs drying until it reaches a moisture level below 15%. Besides the plant structures, the mass of fodder to be dried also influences the speed of dehydration. In other words, the greater the growth and accumulation of dry mass per area, the longer it takes to reach the ideal stage for baling and storage. It occurs when weather conditions do not favor harvesting with excessive and continuous rainfall and high fertilization levels.

Ströher (2015) evaluated the effect of the intensity of damage caused by mower-conditioner with free-swinging flail fingers on the dehydration time of Tifton 85 Bermudagrass and Vaquero plants and obtained dehydrated Tifton 85 Bermudagrass of 4,750 kg/ha with 18% moisture after a dehydration period of 24 h at 21.0–38.5 °C. In Southern Brazil, the dehydration process for ideal storage moisture extends from 96 to 120 h due to the higher incidence of dew and low solar radiation during the winter. In regions with few incident dews, the winter activity favors the reduction of drying time. The more moisture is accumulated on the plant at night, the lower the fodder quality due to the higher respiratory activity of the plant and, consequently, the increase in carbohydrates utilization.

Under good drying conditions, DM losses are between 15% and 18% (Rotz and Abrams, 1988), and with damages caused by rain, values reach up to 30%. In general, hay production processes result in an average loss of 24–28% moisture in fodder DM yield, with the greatest concentration during harvest and approximately 5% at storage (Buckmaster et al., 1989).

In Brazil, the breeding of elite horses has also boosted hay production once the breeding system in stalls requires supplementation with roughage; moreover, silage is not the recommended roughage for this animal species. Equine activity generates R$ 16.15 billion over the year and creates 610,000 direct jobs and 2,430,000 indirect jobs, thus being responsible for three million jobs (MAPA, 2016). The system has evolved by branching into equestrian activities, such as horse racing and equine-assisted therapy, which are in current expansion in the country.

Hay is also used as a physically effective fiber in milk production systems because of its importance on animal nutrition (Mertens, 1997). Besides, hay is sold in agricultural stores as pet feed, such as rabbits and hamsters, and as a theme party decoration. Hay is also used in central Brazil to feed beef cattle, mainly as cylindrical bales of 300 kg (Neres et al., 2017).

This expansion in Central-West Brazil is a result of the replacement of pasture areas by corn and soybean crops for exportation, and the production of pigs and poultry. As swine and poultry farming systems in Brazil are intensive, with high manure production, producers have adopted the use of biodigesters and aerobic ponds for manure. Therefore, biodigested and fermented wastewater from pig farming is now being used in areas planted with hay, and hay fed to beef cattle and the surplus sold out. This system has been adopted in Santa Catarina and the Rio Grande do Sul States.

The weather in the Western Region of Paraná favors the growth of species used in the hay production, and its topography favors mechanization. The greatest challenging months in hay production are those with high rainfall, which delays cutting and consequently compromises the nutritional value of plants. Currently, hay production in Brazil is concentrated in Paraná, Santa Catarina, and the Rio Grande do Sul States where hay drying and storage techniques are practiced. However, the North-East Region (São Paulo, Minas Gerais, and Mato Grosso do Sul States) have seen a significant increase in the number of hay producers who have dedicated themselves to this activity for the past ten years.

The Secretariat of Agriculture and Supply of Paraná (SEAB), through the DERAL (Department of Rural Economy), is responsible for the annual control of hay, silage, and pre-dried hay production in farms of Paraná State. It is the only state in the country that performs this control. According to the information provided by the DERAL/SEAB, the Western Region of Paraná contributed 39% of the state hay production in 2019. Interestingly, hay production in Paraná State increased by 131.4% in the last six years, unlike the production of corn silage that increased by 56.27% (DERAL/SEAB, 2019).

In Southern Brazil, hay production is carried out by corn, soybean, and milk producers. Some farmers specialize in hay production on their property, or leased land, and also provide services to third parties. Those who produce hay and crops reported that the activities do not compete with each other for equipment, resources, and time. These producers adopted the intensive management that annual crops require and apply it to hay areas, including periodic soil analyses, pest and disease control, and soil compaction assessment. In these areas, hay yield per cut in the summer exceeds 4.500 kg/ha.

Milk producers who were already producing hay for animals on the property also began to see the activity as commercially promising and started specializing in the marketing part of their hay and investing in equipment. In other parts of Brazil, hay-producing center's also followed the trend of investing in equipment used in field operations and irrigation.

**Figure 3:**
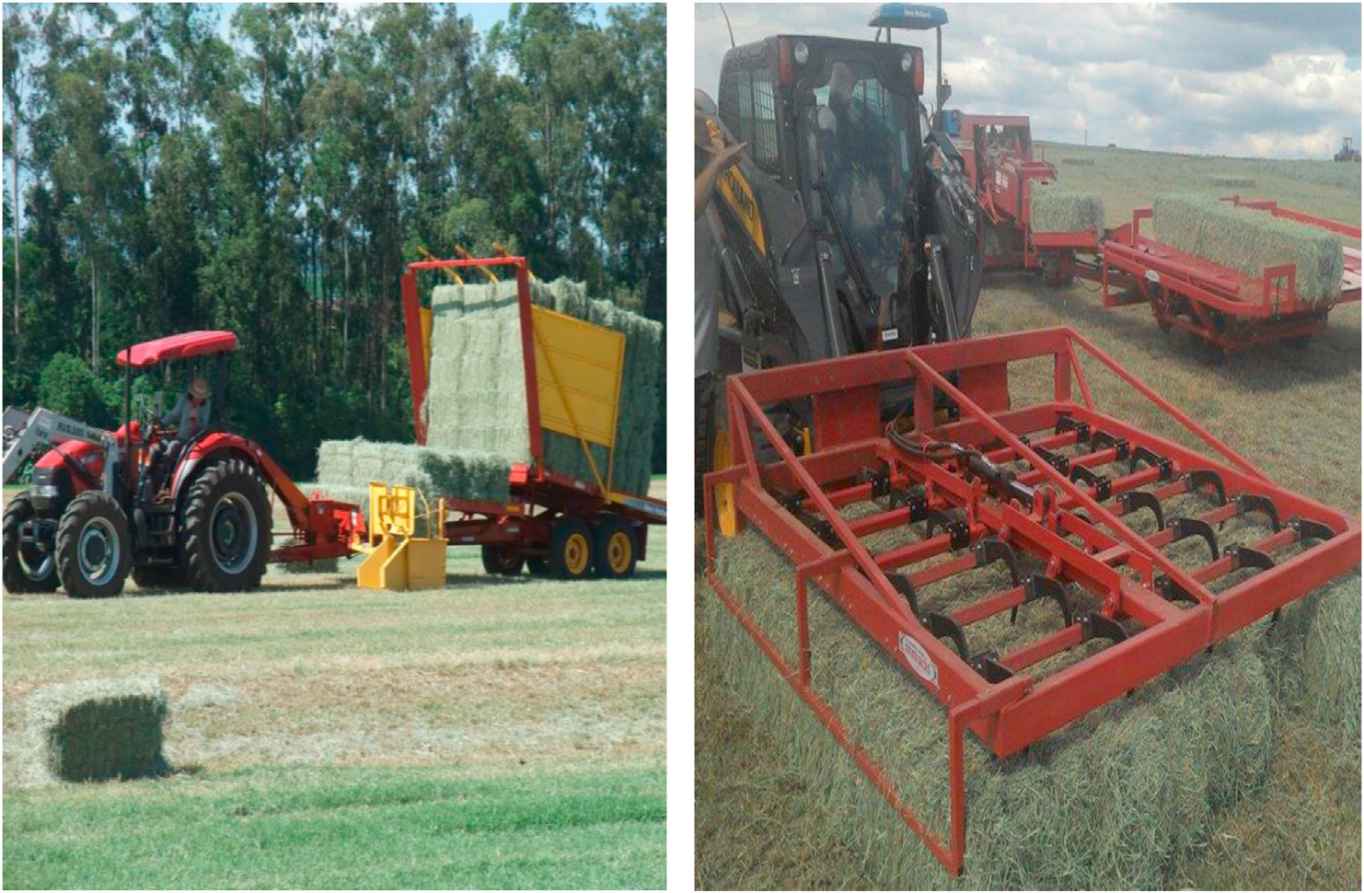
Hay pickers found in the country, imported and national (personal file).

Hay production demands more manpower and a higher frequency of activities than mechanized annual crop production; however, it has some advantages, which the main one is the possibility of having several harvests throughout the year, thus reducing the risk of high-investment and annual crop losses. Moreover, mechanical hay harvesters are now available in markets (Figure 3), and they help to overcome the difficulty of the baling time, which is often unpredictable and may occur on weekends.

In the states where there is no production of animal waste, activity has also been developed using chemical fertilization and fertigation predominately in some properties as fertilization is fundamental in maintaining production systems. In Minas Gerais State, there are hay farmers with production areas larger than 300 ha.

In roughage production areas where harvesting is performed mechanically, there is an intensive extraction of nutrients from the soil, mainly macro elements such as nitrogen and potassium, with little recycling (Sarto et al., 2019). Some producers have invested in center pivot irrigation systems for hay production areas and, to a lesser extent, in subsurface drip fertigation system, which despite the high cost of implementation has the advantage of saving water as it is applied close to the root system, thereby avoiding losses by surface evaporation.

**Figure 4:**
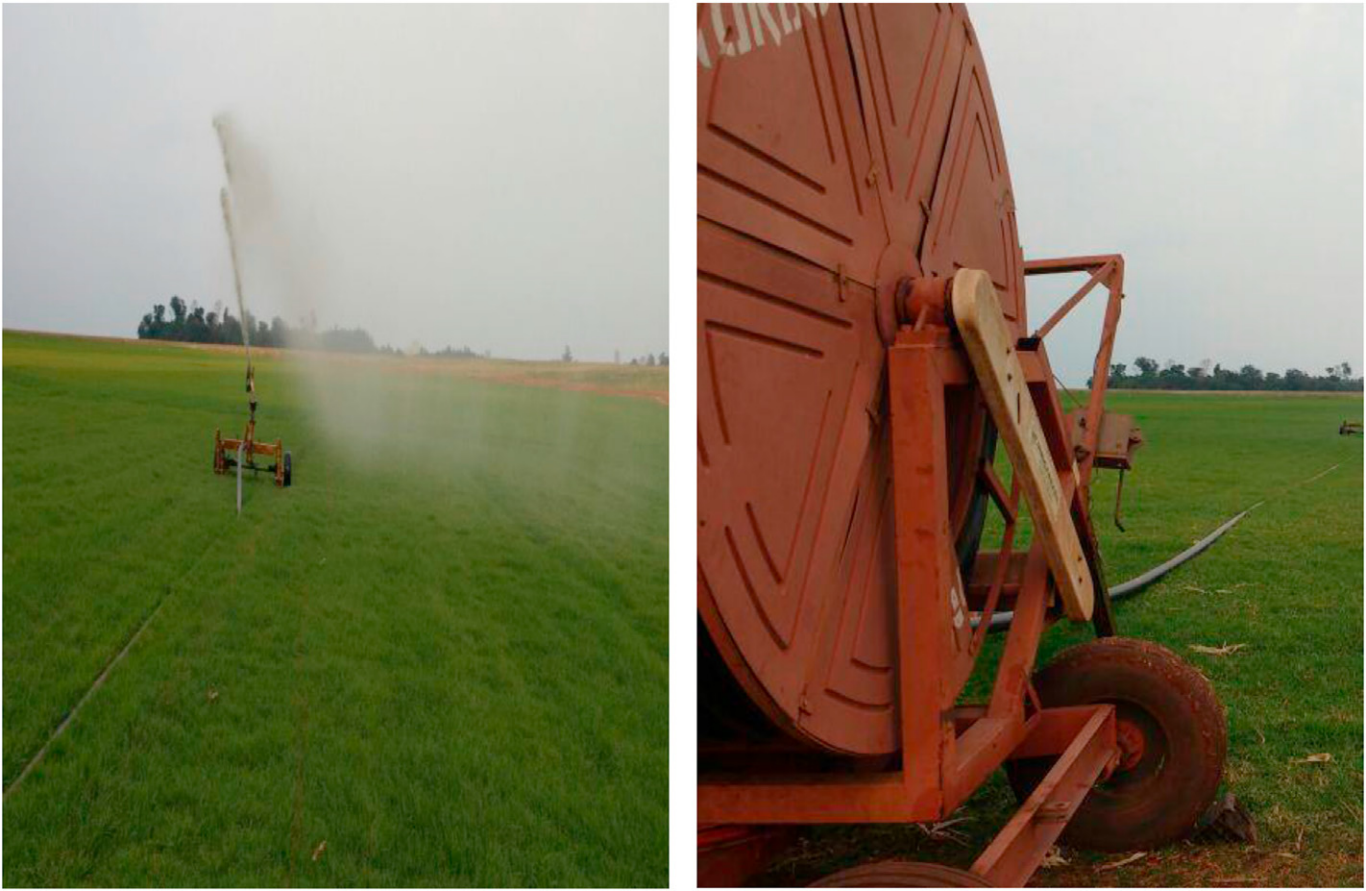
Irrigation of hay producing areas with ARS (swine wastewater) in a reel system (personal file).

In the western region of Paraná, the most common form of fertilization of hay-producing areas is the use of hose reel irrigation of residual watewater from pig farms (Figure 4). Researchers have been monitoring the accumulation of magnesium, zinc, and molybdenum in the soil because these elements, which are part of the pigs' diet, act as prophylaxis of intestinal disorders, replacing antibiotics. The levels of these elements in the soil should be determined annually or biannually, especially in areas where manure comes from UPLs (piglet producing units) because the concentration of these elements in feeds is high, with up to 50% being eliminated through feces.

## 5 Evolution of hay yield research in Brazil

In Brazil, most commercialized hay is from Tifton 85 Bermudagrass, Jiggs, and to a lower extent Vaquero and Coastcross grasses. These are followed by temperate climate grasses such as oats, ryegrass, and alfalfa legume, with the Bandeirantes (Paraná State) as the biggest producer. Hay from the grass of Panicum spp. and Urochloa spp. genera have been also produced.

In preparing an area for the hay production from grasses of the genus *Cynodon* spp., which spread through seedlings, the service, which costs an average of R$2,000.00/ha, is often outsourced and includes soil analysis, fertilization, seedlings, and manpower for planting. The seedlings come from producers specialized in rooted seedlings. Some producers use stolons from their areas to expand their production fields or acquire them from neighbors who have areas planted with the same species.

Tifton 85 grass has the advantage of high nutritional quality and good DM production capacity and is the favorite among hay buyers. It can produce during winter provided that there is no lack of moisture in the soil, and it sprouts up after frost owing to the presence of rhizomes.

Both kinds of grasses, Jiggs and Vaquero, need to be cut at shorter growth intervals to prevent an excessive rise in fiber content after 20 days of regrowth (Ströher, 2015). There remains the problem of balancing the ideal cutting time with the appropriate conditions for processing; it is common for producers to delay cutting owing to continuous rainfall, running the risk of losing the nutritional value of plants (protein content reduction and consequent increase in fiber content). Castagnara et al. (2011) obtained a neutral detergent fiber (NDF) content of 846.10 g/kg in Tifton 85 Bermudagrass at the baling time with 42 days of regrowth due to high rainfall and delayed cutting. These factors result in higher variability of nutritional values among the same hay species, making buyers demand from hay producers a sanitary quality certificate that contains the nutritional composition of the hay. Hay classification tables are usually used in the country, with the classification into types A, B, and C according to CP and NDF values (Table 1).

**Table 1:** Hay nutritional classification in Brazil (Pedreira, 2005).

Type	Moisture	Crude protein (%)	Neutral detergent fiber (%)
A	15–12	>13	<65
B	18–15	9–13	65–69
C	18–15	<9	>69

Prices vary according to season and depend on pasture supply and forage planning. Producers who buy hay in the summer pay a lower price than in winter (Table 2); however, buy it in summer requires having appropriate storage structures to avoid moisture in the bales.

**Table 2:** Tifton 85 and alfalfa (Type A) hay prices in summer and winter/2019 (DERAL/SEAB, 2019).

Hay	Price (R$ kg)	
	Summer	Winter
Tifton 85 Bermuda grass	0.60–0.80	1.00
Alfalfa	1.50	2.50

In regard to storage time, studies by Castagnara et al. (2011), Machado et al. (2019), and Neres et al. (2011) showed that hay from Tifton 85 Bermudagrass and Vaquero grasses does not undergo significant changes in nutritional value for up to 120 days if adequately stored. Further studies are needed to evaluate storage times longer than six months.

About winter fodder, black oat is one of the most used temperate climate grasses for hay production in southern Brazil. Black oat has thicker stalks and needs more time between 124 and 144 h for adequate dehydration (Castagnara et al., 2012). The use of mower-conditioners for these forage plants allows earlier baling.

Overseeding annual winter forages on Tifton 85 Bermudagrass hay production areas in southern Brazil and the São Paulo State is also a technique used by hay producers to maximize hay production in winter once there is a reduction in mass production of tropical fodder (Neres and Ames, 2016). However, this technique has the disadvantage of reducing the regrowth power of Tifton 85 Bermudagrass in early spring (Ames et al., 2014) owing to plant competition, shading, and water and nutrient extraction, which delays initial cuts in periods favorable for the production of hay from tropical fodder (Neres et al., 2011). Oat overseeded on Tifton 85 Bermudagrass hay production areas tends to suppress the first spring cut of Tifton 85, making many producers opt for growing the species in separate areas.

Alfalfa has a higher market price than C4 grasses owing to its lower mass production and crop management costs, and because the hay has a more restricted market composed mainly of breeders of elite animals, it takes higher market prices because of higher production costs (Neres et al., 2011). Researchers face a challenge in the production of pre-dried alfalfa because of its low DM content at the time of cutting, high buffering power, and low soluble carbohydrate content, which reduce sanitary quality and cause rapid deterioration of roughage product.

**Figure 5:**
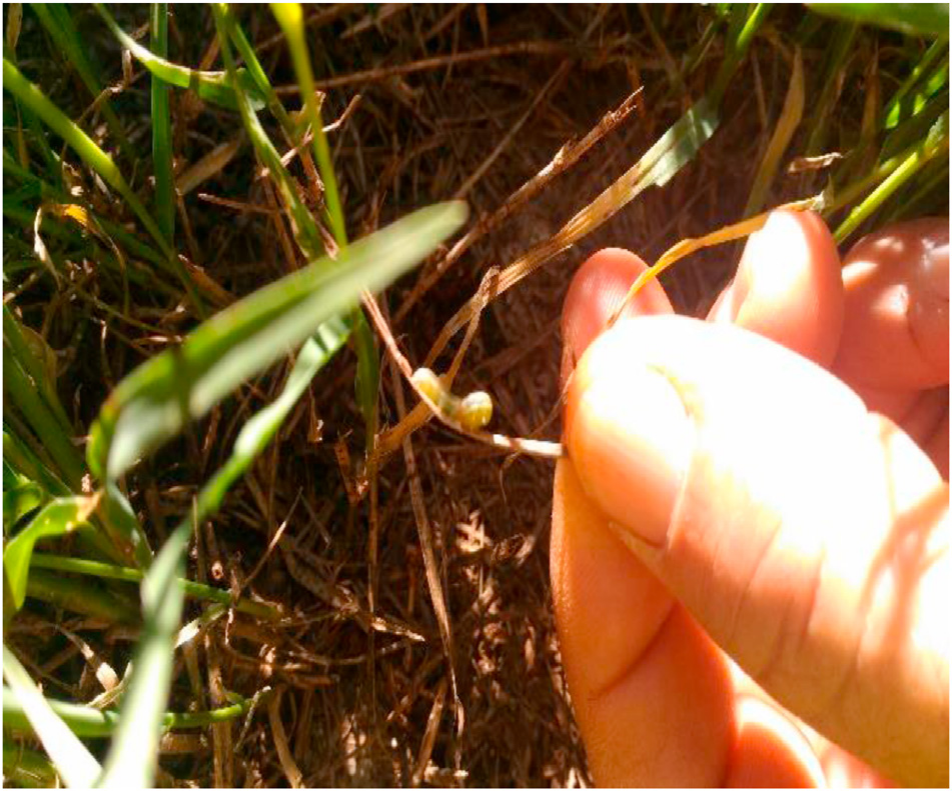
Caterpillar infestation in a Tifton 85 hay production area (personal file).

The main economically important pests that usually infest alfalfa and Tifton 85 Bermudagrass are pasture leafhopper and corn caterpillar (Figure 5). The oat's main disease is rust and is usually more intense in white oat and more frequent at the end of the cycle. These pests: *Mocis latipes*, *Spodoptera fugiperda,* and *Deois flavopicta* and diseases vary in intensity of effects according to prevailing weather conditions.

## 6 Equipment used in hay production

Cutting of forage for haymaking is performed using a drum or knife mowers. Currently, the number of producers who choose to purchase knife mower-conditioners is increasing because hay production requires specific climatic conditions, such as high solar radiation, absence of rainfall, and low relative humidity (Pasqualotto et al., 2015). The use of mower-conditioners also allows early storage of hay because the damage caused by the machine on the cuticle of plants at the time of cutting help to accelerate the dehydration process, especially in the stems and culms during the second stage of dehydration, when perspiration occurs via the cuticle (MacDonald and Clark, 1987).

However, under rainy conditions, the mower-conditioner utilization increases DM losses compared to the conventional mowers (Rotz, 1995). Pasqualotto et al. (2015) obtained high rates of dehydration with high conditioning intensity (obtained by adjusting the deflection plate) in the first 24 h after cutting.

In the studies by Machado et al. (2019) and Neres et al. (2011) with Tifton 85 Bermudagrass and Vaquero grasses, under high solar radiation and winds, the use of the conditioner did not take more than 2 h in windrowing and baling of fodder. During autumn/winter, the conditioner effect is more pronounced, especially on oat because it has thicker stems than Tifton 85 Bermudagrass.

**Figure 6:**
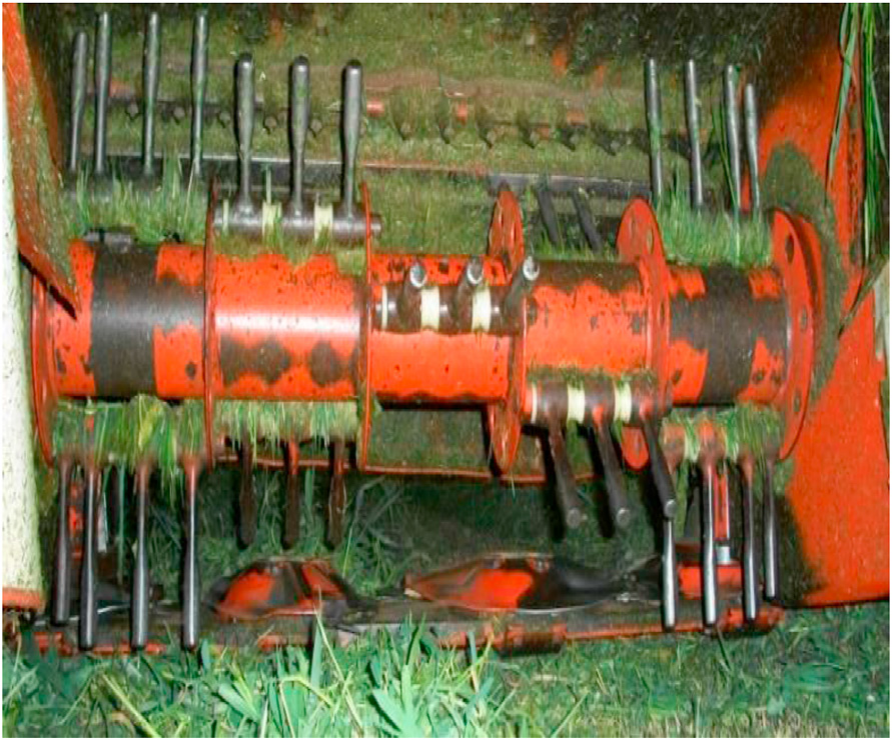
Conditioner mower with free-swinging flail fingers (personal file).

Most of the mower-conditioners used in Brazil have iron-free-swinging flail fingers (Figure 6), and mowers used for legumes have rubber rollers. The iron free-swinging flail finger mower-conditioners damage intensity is regulated by adjusting the distance of the deflection plates or the rotation speed, ranging from 600 to 1000 rpm (Pasqualotto et al., 2015). In Brazil, the trade of mower-conditioners with flails is high owing to the predominance of grass hay, which has greater yields and leaf loss.

After cutting, the high amount of water in the plant promotes a dense layer formation with low air circulation within the hay. Turning should be performed with a rake to promote ventilation inside the windrow. Under tropical conditions, turning is performed at least twice in the spring/summer, between the first and second drying stages.

The height of the rake must be adjusted to prevent the entrance of soil organic matter into the newly cut hay. Studies by Schmoeller (2016) highlight the high incidence of zearalenone mycotoxin (ZEN) in plant material deposited on the ground in areas of Tifton 85 Bermudagrass and Vaquero grass hay production (Table 3). ZEN is an important mycotoxin produced by *Fusarium* spp. and causes significant damage because it is a potent estrogenic metabolite (Kumar et al., 2008).

**Table 3:** Averages and standard deviations (in parenthesis) of aflatoxins (AFLA), zearalenone (ZEA) and deoxynivalenol (DON) according to the storage method in Tifton 85 and Vaquero Bermuda grass production areas (Schmoeller, 2016).

	AFLA (mg/kg)	ZEA (mg/kg)	DON (μg/kg)
Dead plant matter	0.10^c^ (0.15)	311.4^a^ (319.2)	0.013^c^ (0.04)
Bermudagrass in baling	0.25^c^ (0.32)	15.4^c^ (8.84)	0^c^ (0)
Hay in covered shed	2.83^a^ (0.77)	74.8^b^ (42.2)	0.24^a^ (0.07)
Hay in transparent canvas	2.68^ab^ (1.43)	85.9^b^ (28.9)	0.18^b^ (0.07)
Hay in double-sided coated canvas	1.85^b^ (0.61)	84.2^b^ (41.1)	0.05^c^ (0.08)

Means followed by different lower case letters in the column differ from one another by testing the difference in *lsmeans*, at a 5% probability level; n = 8; p≥χ^2^_(ENFxDF) (DON)_ = 0.0603.

It is common in Brazil not to windrow hay at night because cut fodder tends to dry in the first hours (Calixto Jú nior et al., 2007). After reaching a moisture level below 15%, the fodder is ready to be windrowed with a hay rake.

Pasqualotto et al. (2015) evaluated the rates of net oxygen assimilation, stomatal conductance, and perspiration using an infrared gas analyzer (IRGA) (6400 xt) in Tifton 85 Bermudagrass, cut with a mower-conditioner under tropical conditions. They observed that 4 h after harvest, under the testing conditions, much plant water had been loose via stomata transpiration (*E*) and cuticular transpiration (60% DM); the transpiration values of plants cut under low conditioning intensity were lower (55%) than those of plants cut under high intensity (Table 4). Nevertheless, the DM content for baling (80.40%) is achieved after 49 h of dehydration.

**Table 4:** Evaluation of liquid assimilation, stomata conduction and transpiration of Tifton 85 Bermuda grass before cutting and in the first four hours of dehydration with high (8 cm) and low (18 cm) intensity of conditioning∗ (Pasqualotto et al., 2015).

Intensity	Before cutting	1 h	2 h	3 h	4 h
	Net assimilation (μmol CO_2_ m^2^/s)				
High	22.46a	-2.46bA	-2.72bA	-2.32bA	-2.58bA
Low	22.46a	-5.29bA	-5.71bA	-5.53bA	-4.99bA
	Stomatal conductance (mol m^2^/s)				
High	0.115c	0.093cA	0.067cA	0.27aA	0.22bA
Low	0.115a	0.069aA	0.067aA	0.068aB	0.065aB
	Transpiration (mmol H_2_O m^2^/s)				
High	1.77b	2.09bA	1.86bA	4.49aA	4.63aA
Low	1.77a	1.88aA	1.95aA	2.13aB	1.92aB

Averages followed by lowercase and uppercase on the column do not differ significantly among themselves by Tukey's test at 5% of significance. ∗ Distance from the deflection plates.

Hay buyers still highly prefer the small prismatic bales (10–20 kg), but the balers currently available on the market are diverse. The choice of bale weight and shape is related to the availability of equipment on the property or workforce. Round balers were developed in the 1970s (Freixial and Alpendre, 2013) and have been used in Brazil in recent years. The greatest advantage of the large round baler for hay producers is the amount of time saved in hay baling.

The round balers in the market have a fixed, variable, or semi-variable chamber. What differentiates them is that bale compression occurs as hay enters this chamber in the variable chamber baler but only after the end of hay harvesting in the fixed chamber baler. Further studies on the effects of round baler fodder compression systems on the quality and durability of conserved fodder are needed.

Currently, there are round balers that chop fodders in the field at the time of harvesting (optional). These balers have knife blades (eight to 26 blades with adjustable chopping width and a varying length ranging from 4 cm to 20 cm) and are currently preferred by hay and pre-dried hay buyers. In this case, it is necessary to use plastic nets to wrap the bales instead of straw or plastic string to avoid physical losses of material.

## 7 Outsourcing in the production of hay and pre-dried hay in Brazil

Producers who grow corn for silage intended for animal consumption on medium to large properties tend to follow the trend of outsourcing the harvesting, opting for self-propelled harvesters. Many hay growers start production by outsourcing the production activity, unlike corn silage producers. Hay growers need to acquire their implements owing to the nature of hay production activity, which depends on favorable weather windows, and is carried out 6 to 8 times a year, practically with no margin for waiting; conversely, corn silage productions have one harvest and in some regions one harvest and/or an interim harvest (Neres et al., 2017).

Capitalization of hay producers, short weather windows favorable for drying, and the difficulty in obtaining a temporary workforce for baling, storing, and loading for sale have led producers to opt for the purchase of implements with greater efficiency and yield. The investments are high, considering that haymaking requires mowers, rakes, and balers. The main objective of purchasing more efficient equipment is to reduce the time spent in various hay production and pre-drying activities (cutting, turning, baling, and storage) and thus minimize weather-related losses.

The amount paid for outsourced service corresponds to 50% of the hay costs. Many machinery owners choose to receive in national currency, which corresponds to 1/3 of the commercial hay value. These service providers have no interest in the commodity or structures for storage.

## 8 Product storage and transportation

Hay that is not immediately commercialized should be stored in covered ventilated sheds, protected from the rain. Wooden or plastic pallets should be placed on the floor to prevent direct contact between hay and floor and moisture changes. Commercial hay producers store hay using a storage system when there is excess production during summer so that the market value will increase in times when supplementation with roughage is scarce, that is when supply is low and demand is high. In this system, the storage structures are concrete sheds with aluminum roofing and ventilation.

**Figure 7:**
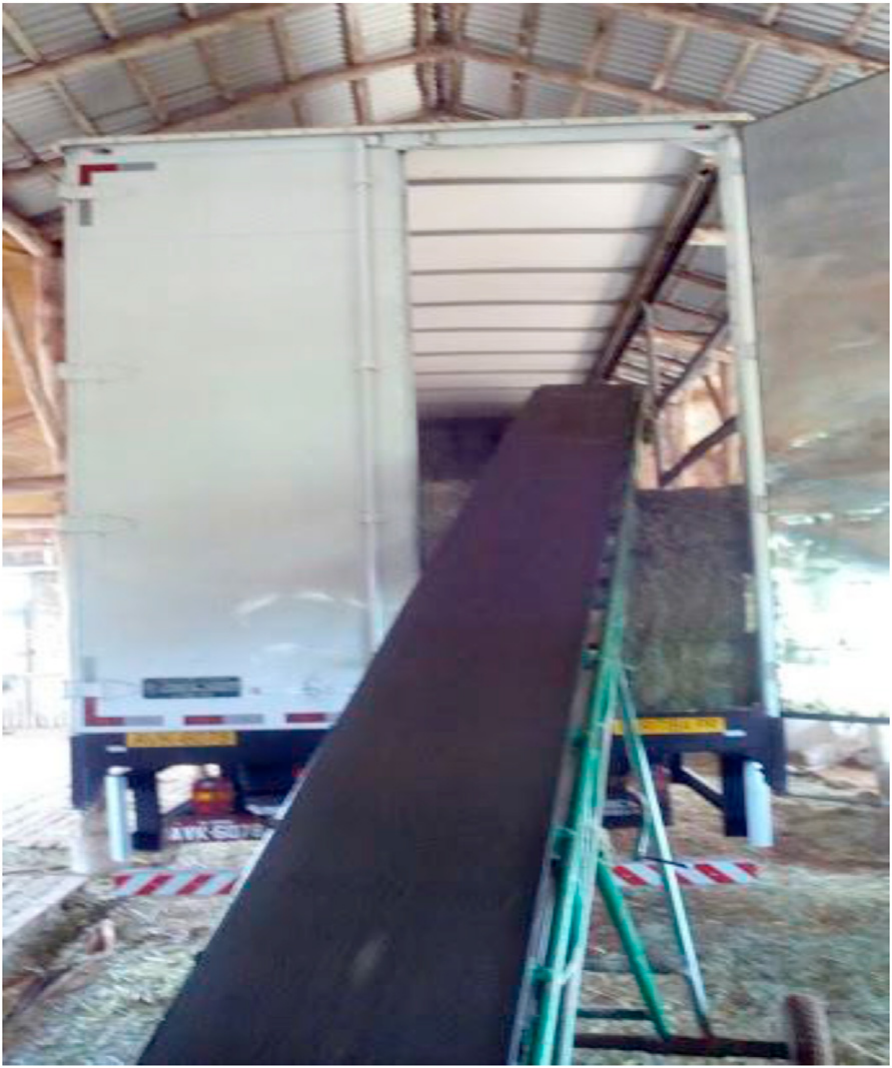
Loading hay for sale (personal file).

The profitability of selling hay to other cities and states is dependent on transportation, done mainly by trucks. Freight, paid by the buyer, is currently around R$ 2.80 and 3.00/km. Many hay growers sign contracts with horse or cattle breeders for a fixed monthly supply of hay. The advantage is that there is a fixed demand; however, in periods of unfavorable weather conditions for the fodder growth, the hay producer is forced to comply with the conditions stipulated in the contract and often needs to buy the product from third parties in the absence of stock. The hay should be transported in closed trucks or protected with tarpaulins to prevent rain and moisture from entering the bales (Figure 7). The trucks carry an average of 1,400 bales of 20 kg. The price of freight often makes it impossible to sell hay to distant markets.

Producers in the country are beginning to view hay exportation as a new niche market, with some producers exporting to the Arab Emirates.

## 9 Conclusion

Hay-producing centers and investment in hay equipment and technology have been growing in Brazil due to the increased use of roughage in the diet of several species and categories of animals and its economic return. This growth has resulted in the expansion of a new market niche, which has the potential to be known in the international markets. The scientific papers related to Brazilian's hay yield point to the high potential to plant dehydration in summer/spring, associated with the ideal storage conditions, result in the maintenance of nutritional and sanitary qualities for about six months. The use of conditioner mowers anticipates the hay dehydration period, which avoids losses by rainfall and, also, anticipates the bailing process. The haymaking in Brazil contributes as an extra income source to producers, the external market opening, and the residual water use from biodigesters contributes to sustainable production. Brazilian haymaking and commercialization are in an expansion process with economic return as national and international trade. Further challenges: to obtain a constant annual hay supply and the transport viability to markets distant from the production center.

## Declarations

### Author contribution statement

All authors listed have significantly contributed to the development and the writing of this article.

### Funding statement

This research did not receive any specific grant from funding agencies in the public, commercial, or not-for-profit sectors.

### Data availability statement

Data included in article/supplementary material/referenced in article.

### Declaration of interests statement

The authors declare no conflict of interest.

### Additional information

No additional information is available for this paper.
